# Case Report: Radiopharmaceutical extravasation, radiation paranoia, and chilling effect

**DOI:** 10.3389/fnume.2024.1349527

**Published:** 2024-02-05

**Authors:** Jason Mace, Jackson W. Kiser

**Affiliations:** Department of Molecular Imaging, Carilion Clinic, Roanoke, VA, United States

**Keywords:** radiopharmaceutical extravasation, SNMMI, diagnostic imaging, medical event reporting, radiation paranoia

## Abstract

The Society of Nuclear Medicine and Molecular Imaging (SNMMI) has publicly commented that they do not support the reporting of large extravasations to patients or regulatory bodies. The comment cites recently published articles suggesting that extravasations are infrequent and not severe. The comment stresses the importance of ensuring patients are not apprehensive or resistant to nuclear medicine procedures because of “radiation paranoia” and a “chilling effect” that can result from misinformation. Radiation paranoia and chilling effect are not defined, and there are no references to specific misinformation. Our experiences and this case suggest the comment may be incongruent with real-world clinical experiences. Our severe case, at a center with a long-standing focus on reducing radiopharmaceutical extravasation, suggests these events can still happen, can be significant, and should be shared with our patients. Our experiences also suggest that being transparent with patients builds trust. We are concerned that a reluctance to recognize the true frequency of extravasations and their severity may create distrust in the relationship between the nuclear medicine community and patients.

## 1 Introduction

On September 1, 2023, the Society of Nuclear Medicine and Molecular Imaging (SNMMI) submitted a public comment (the comment) to the U.S. Nuclear Regulatory Commission (NRC) regarding the reporting of radiopharmaceutical extravasations as medical events (1). In their comment, SNMMI stated:

“The safety of our patients and the highest quality of care are our top priorities. We also must ensure that patients who would benefit from nuclear medicine procedures are not apprehensive or resistant to safe, often lifesaving procedures because of “radiation paranoia” or a “chilling effect” that can result from misinformation. We support harm based, rather than dose-based approach, as has been recommended by the NRC.”

The comment does not define the conditions of “radiation paranoia,” “chilling effect,” or the specific misinformation that may lead to these conditions. The comment does, however, imply that misinformation is driving the NRC to consider reporting extravasations that meet the same dose-based threshold used to consider other reportable medical events. To support the position that NRC should wait for patients to experience deterministic effects before an extravasation should be considered reportable, the comment cited two recent studies as evidence of “both the infrequency and lack of severity of extravasations in nuclear medicine.”

To support the claim that extravasations are infrequent, the comment cited a study based solely on a review of radiology reports. However, prior research had already concluded that, due to the nature of radiopharmaceuticals and methods of administration, most radiopharmaceutical extravasations are unnoticed by clinicians and patients; and that even when visible, they are rarely included in the radiology report (2, 3).

To support the claim that extravasations are not severe, the comment cited this same study and a second study that retrospectively reviewed PET/CT images of 1,000 patients and, through imaging and Monte Carlo estimates, attempted to assess the risk of radiation to skin and tissue. This second study reported that the amount of radioactivity in the six most severe extravasations was between 6 and 50 uCi and that, based on hypothesized lateral distribution of radioactivity in the hypodermis, the sensitive epidermis is spared. The authors concluded that the “risk of skin injury is significantly lower than implied in the current literature…” and suggest their conclusions are confirmed by lack of observable skin effects over decades of nuclear medicine procedures. The authors do not address the impact of radiation dose to the hypodermis or underlying muscle tissue. They do not address literature that counters lateral distribution of the extravasated radioactivity (4). They do not address extravasations of large amounts of radioactivity. And they do not address the lack of long-term patient follow-up looking for skin or underlying tissue damage caused by extravasations.

Even though the SNMMI had previously taken the public position that extravasations can impact the quality and quantification of diagnostic images, the comment opposed requiring a device to detect extravasations (5). Even though SNMMI practice guidelines endorse assessing the severity of extravasations (6) the comments to NRC opposed assessing extravasations. When the NRC specifically asked what information licensees should provide to patients on how to identify an extravasation and what to do if a radiation injury is suspected, the comment simply stated: “Patients receiving diagnostic radiopharmaceuticals need not be concerned.”

Carilion Clinic is a non-profit healthcare system located in southwest Virginia. We are committed to providing an environment that fosters quality health care and respect for each individual patient. We believe it is important for our team to share information with patients, both because they have the right to know about their treatments, and so that they can make informed decisions about their care.

Based on our review of the literature and our own experiences, we have reached the following conclusions about radiopharmaceutical extravasations. There is incontrovertible evidence that large extravasations (more than a nominal percent of the injected activity) can negatively affect the quality and quantification of images that help determine and guide the care of our patients (7–12). Likewise, there is evidence that absorbed radiation doses arising from radiopharmaceutical extravasations (even diagnostic radiopharmaceuticals) can be much higher than an absorbed dose of 1.0 Gy, a threshold the SNMMI has previously said can lead to deterministic effects (13–15). Furthermore, it is well established that radiosensitivity varies among individuals, and that our patient population in nuclear medicine is more radiosensitive than healthy individuals (16).

Our experiences are consistent with the literature. For large diagnostic radiopharmaceutical extravasations, we have calculated absorbed radiation doses greater than 1 Gy–5 cc of tissue in many patients. Isotopes associated with these radiopharmaceuticals include 18F (which locally deposits energy in the tissue from positron emissions) and 99mTc (which locally deposits energy from internal conversion electrons, auger electrons, and low energy x-rays). While there are many who suggest that low doses of radiation are not dangerous, there is little debate that large radiation doses to healthy tissue are not in the best interest of patients.

As part of our effort to provide quality health care, our teams have undertaken continuous efforts to reduce the frequency and severity of these events at our facilities since November 2016. To this end, we have prospectively monitored radiopharmaceutical administrations with the latest technology available. While we have shown significant reductions in extravasation rates, we have not eliminated them entirely. When we know, through our monitoring efforts, that a patient has been extravasated, we follow a standard response. We include the injection site in the imaging field of view, and we use data from our monitoring sensors and the images to calculate absorbed dose to 5 cc of tissue. We also quantitatively assess the impact of the extravasation on the procedure, we document and share this information with the patient, and, as needed and in consultation with their physician, we reschedule patients for repeat procedures at our expense.

Six weeks after the comment was submitted to the NRC, we encountered an extravasation of 18F-FDG at one of our satellite nuclear medicine facilities. This mobile PET/CT center has been an active participant in our multi-year efforts to improve the quality of radiopharmaceutical administrations and has seen steady and statistically significant improvements. This case has regulatory implications. It shows that even at facilities focused on reducing these misadministrations, severe extravasations involving large amounts of radioactivity can still occur, and there is a need for physician and patient concern.

### Case presentation

A male (>70 years old) patient was initially diagnosed with Non-Hodgkin lymphoma in September 2020 and underwent treatment at that time. He experienced a recurrence in June 2021 and was re-treated successfully. A follow-up PET/CT in April 2022 showed another area of recurrence, and he underwent additional re-treatment. The patient has been disease-free since that time. In October 2023, he presented for a routine treatment monitoring PET/CT study.

**Figure 1:**
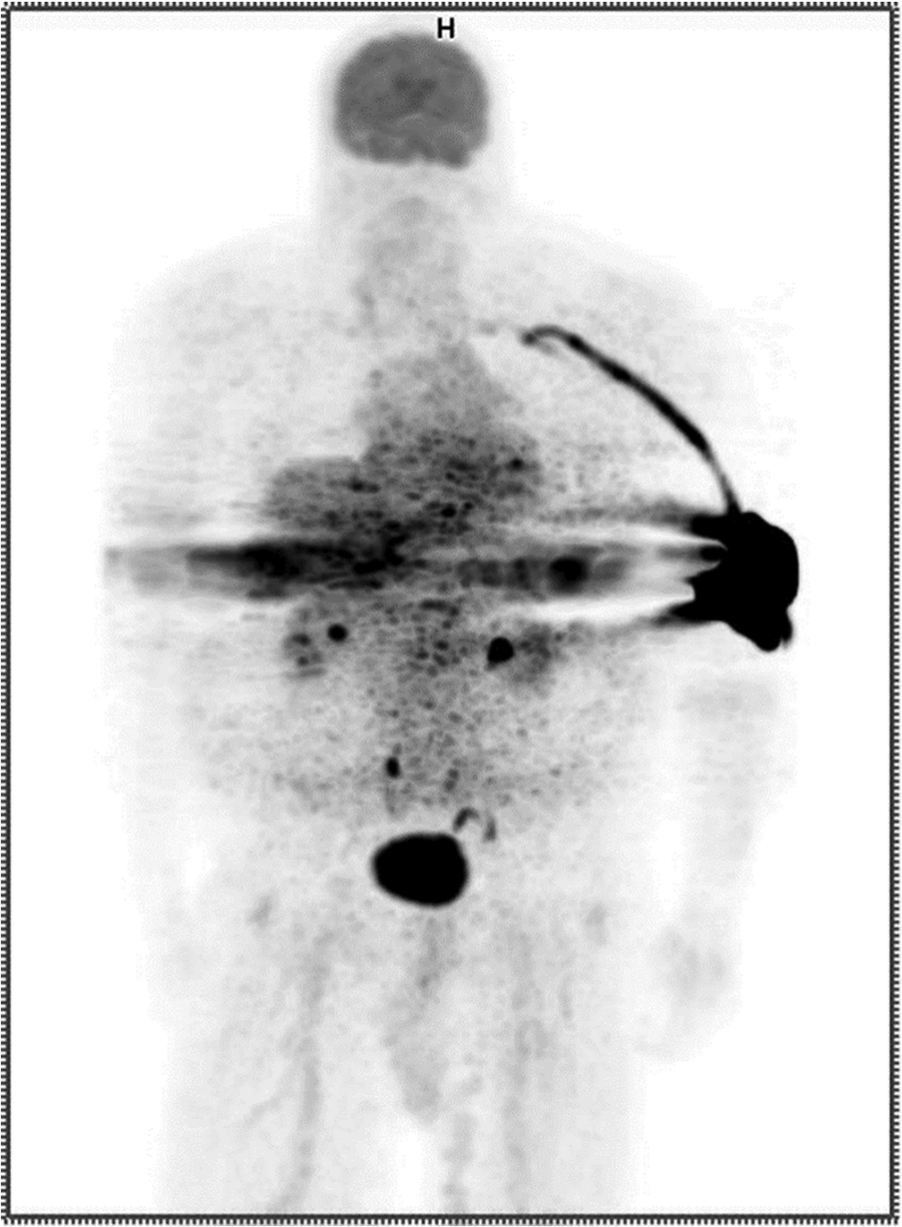
Anterior maximum intensity projection (MIP) PET image showing extravasation in the patient's left antecubital fossa.

A certified nuclear medicine technologist established venous access using a 22 gauge IV in the patient's left antecubital fossa and noted blood return. The technologist administered 12.09 mCi of 18F-FDG as a bolus injection and flushed the IV with 20 cc of saline. Readings from our external monitor positioned proximal to the injection site on the patient's arm indicated radioactivity may have remained in the vicinity following completion of the administration. Given this evidence that the patient might have been extravasated, the patient's arms were included in field of view during imaging. The resulting image was non-diagnostic. (Figure 1) Our technologists explained to the patient they had been inadvertently extravasated and asked the patient if he had experienced any discomfort during the administration. The patient responded he did not notice anything unusual during the administration of the radiopharmaceutical but may have felt a slight burning sensation during the saline flush. The technologist shared that the imaging procedure might need to be repeated and that they would reach out to the patient with more information. They also told the patient that if symptoms develop at the injection site, he should report them.

**Figure 2:**
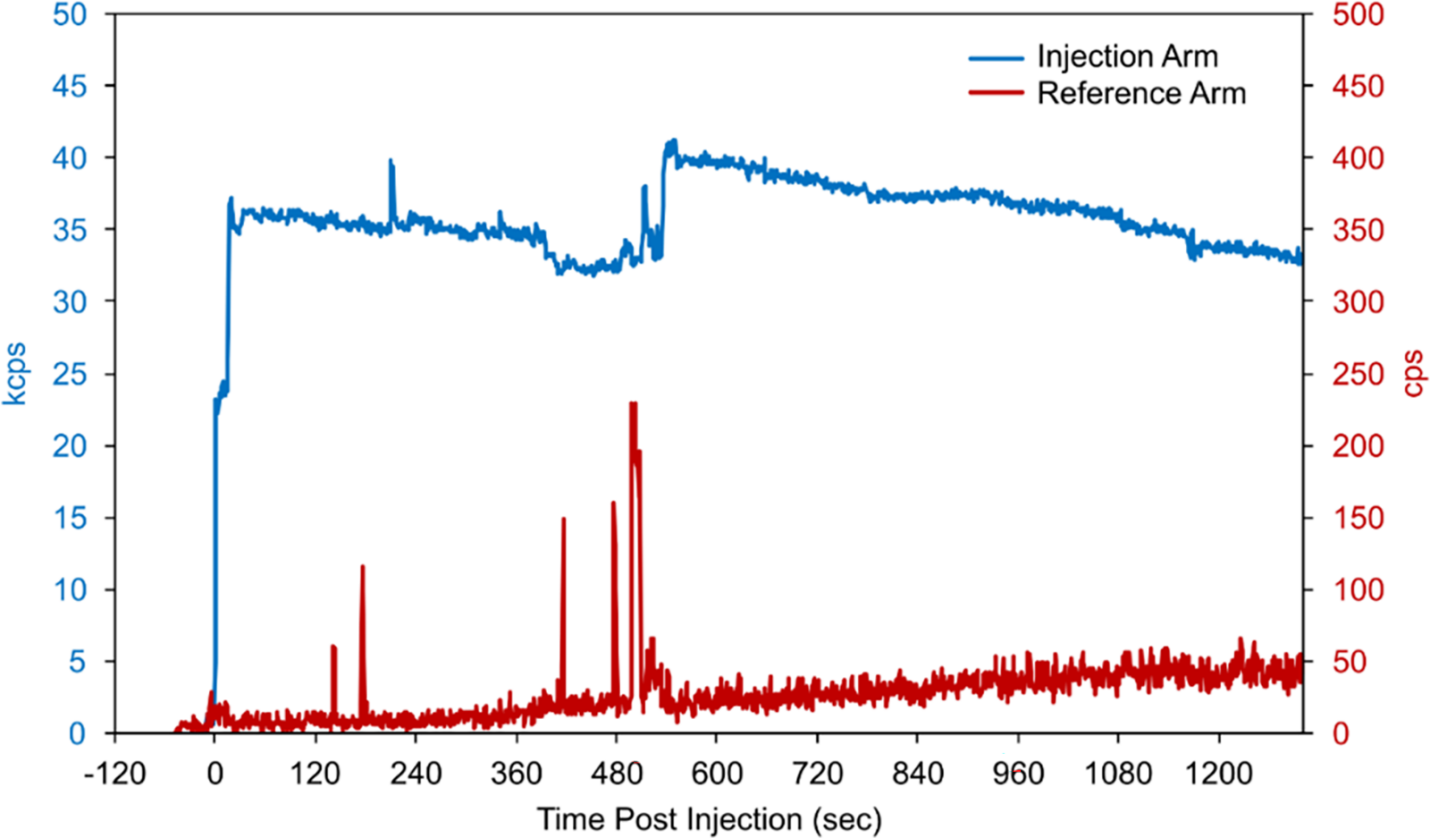
Count-rate data recorded by monitoring sensors placed on the patient's biceps during and following bolus injection. When compared to the reference-arm sensor counts (red, secondary y-axis), the persistently elevated counts from the injection-arm sensor (blue, primary y-axis) indicate the likely presence of residual radioactivity.

Based on biological clearance data from the monitoring sensors (Figure 2) and imaging information, our Radiation Safety Officer calculated an absorbed dose of 17.3 Gy to the affected tissue using previously published methods (17). The event was documented in their medical record and reported to the radiation safety committee. The patient underwent a free repeat imaging procedure one week later.

A timeline of the events and proposed patient follow up is included (Table 1).

**Table 1:** Timeline of the extravasation event and planned follow-up.

2023		Timeline Post-Procedure			
October		1–2 weeks	1–2 months	3–4 months	6–9 months
Exam 1. Extravasated	Exam 2. Repeat Imaging	Evaluations to assess any potential tissue or skin effects			
Dosimetry Performed					

The patient consented to the use of their de-identified images for publication and presentation for educational purposes consistent with the Carilion consent process.

### Diagnostic assessment

**Figure 3:**
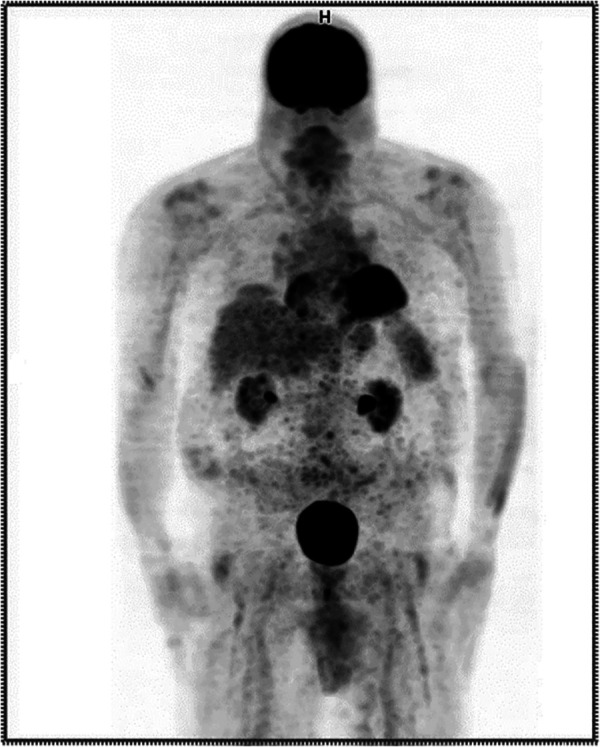
Anterior maximum intensity projection (MIP) PET image from the repeated procedure showing no evidence of extravasation.

The repeated PET/CT study (Figure 3) indicated no active disease.

## Discussion

Based on our seven years of experience closely monitoring for extravasations in over 17,000 diagnostic and over 175 therapeutic nuclear medicine procedures at several sites, the evidence cited in support of the comment does not reflect reality. Without effective monitoring of each administration (either prospectively or retrospectively), an NRC licensee cannot accurately assess the frequency of their extravasations. Without effective monitoring and assessment of large extravasations, nuclear medicine centers will not know which extravasations have a potential to negatively affect patients, either through deterministic radiation effects or due to the impact on the procedure.

**Figure 4:**
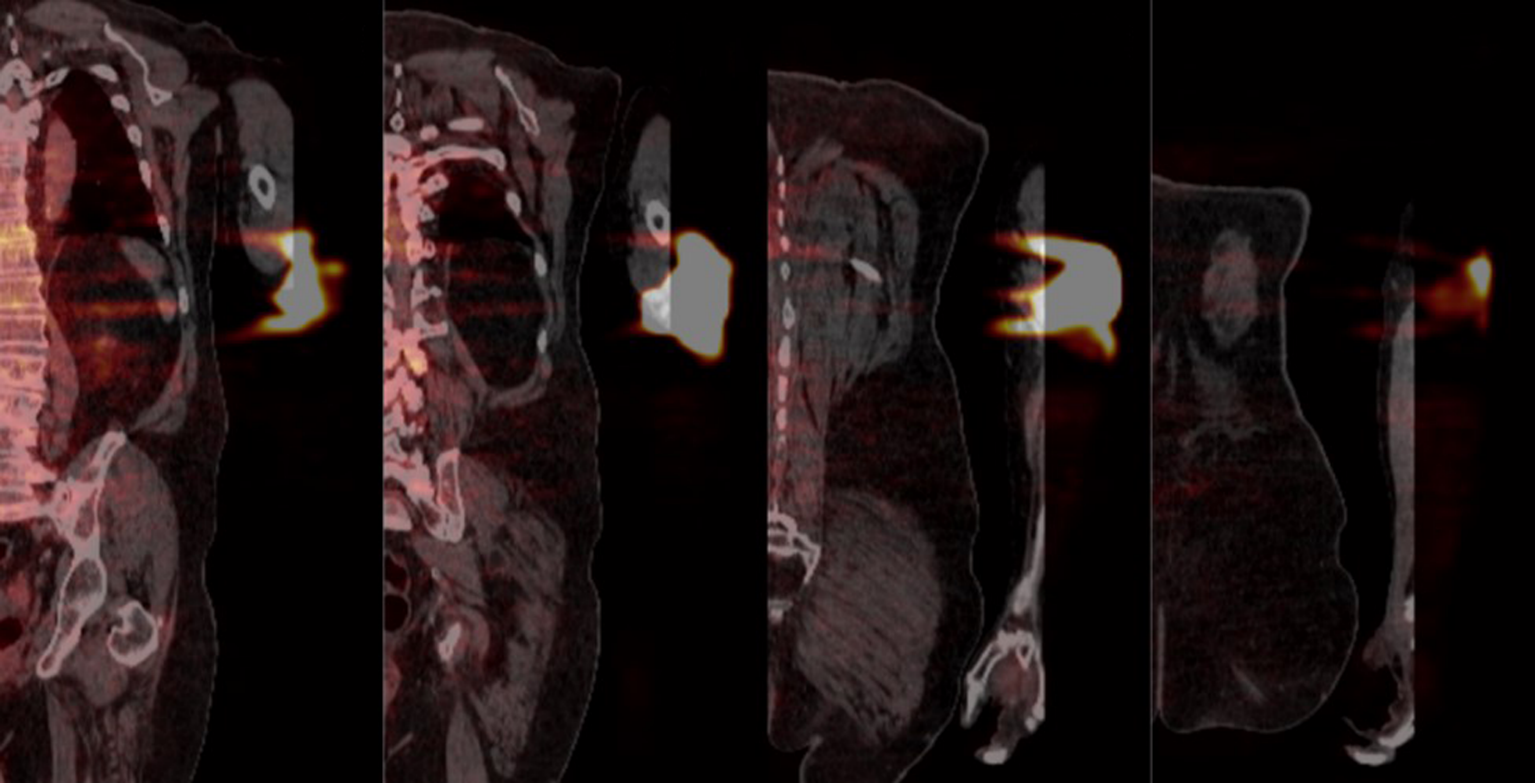
Coronal PET/CT views from anterior to posterior (left to right respectively) detailing the extravasated area. The distance between successive slices shown is approximately 40 mm.

We have found that active monitoring can help reduce both the frequency and severity of extravasations. Additionally, we have learned that when an extravasation does happen, it is important to assess the severity of the extravasation. The suggestion, highlighted in the comment, that extravasated radioactivity remains compartmentalized in the hypodermis is biologically implausible, irrelevant, and contradicts our observed experiences. While we understand the limitation of the coronal views in this case (Figure 4), there is little indication that radioactivity was only distributed laterally in the hypodermis. And even if that were the case, the adjacent layer of dermis, the hypodermis, the adjacent layer of connective tissue, and muscle would be irradiated with energy. Not only does assessing the severity of the extravasations help us estimate the absorbed dose to tissue and overlying skin, but assessing the radioactivity that was not initially distributed systemically as a bolus also helps us assess the effect of the extravasation on the image.

When a radiopharmaceutical administration results in a large extravasation, we strive for complete transparency with the patient and their treating physician. Our approach has enhanced the trust of our patients. At Carilion, we do not report these rare, large extravasations to any regulatory agencies because there is no such reporting requirement at this time. However, an honest assessment and review of contributing factors associated with these cases helps us understand why they occur and feeds our improvement processes. A reduced extravasation rate is good for patients, for us, and for nuclear medicine.

Our approach to extravasations is entirely consistent with our mission and belief system at Carilion. While we can still improve how we address radiopharmaceutical extravasations and how to clinically follow affected patients, we believe that our current extravasation policy helps to ensure that our patients receive the same care and attention we would want for our families and ourselves.

## Concluding remarks

The SNMMI has long argued against the reporting of radiopharmaceutical extravasations. Most recently, they have stated that they are concerned that patients will forgo important nuclear medicine procedures because of “radiation paranoia” caused by misinformation. This statement and their past arguments are not supported by our real-world experience.

Paranoia is “unjustified suspicion and mistrust of other people or their actions.” We have studied the causes of extravasations. Extravasations are almost entirely preventable. The inadvertent injection of radiation into a patient's tissue can result in unnecessary irradiation and result in doses that easily exceed current reporting requirements. These extravasations also negatively affect the diagnostic imaging procedures that guide patient care. The preventability and consequences of extravasations are supported by numerous peer-reviewed publications. This is not misinformation. And since reporting is intended to reduce preventable accidental radiation exposures that exceed the dose-based threshold, the current inconsistency in reporting requirements for accidental irradiation of patients should be addressed. We see no scientific justification for holding extravasations to a different standard than other medical events. Providing licensees with an appropriate grace period to address the factors that lead to extravasations and then mandating reports of large extravasations will ensure that licensees are providing the radiation protection and transparency to patients that is needed.

In our experiences, being transparent with our patients and our referring physicians in the event of an extravasation enhances trust. If there is concern that facts about extravasations will lead to a chilling effect that we assume refers to procedural volume, we have not seen that. Our extravasated patients have appreciated our approach. We have not seen apprehension, hesitancy, or resistance to receiving further procedures. However, if our community acts on the suggestion that patients should not be concerned when they have experienced a large diagnostic radiopharmaceutical extravasation, that will lead patients to mistrust our community. And that may have a chilling effect that is dangerous to patients and to our profession.

## Data Availability

The raw data supporting the conclusions of this article will be made available by the authors, without undue reservation.
